# Can thiol-based redox systems be utilized as parts for synthetic biology applications?

**DOI:** 10.1080/13510002.2021.1966183

**Published:** 2021-08-11

**Authors:** Ché S. Pillay, Nolyn John

**Affiliations:** School of Life Sciences, University of KwaZulu-Natal, Pietermaritzburg, South Africa

**Keywords:** redox signaling, synthetic biology‌, redox systems biology, reactive oxygen species, peroxide, redox sensors, redoxin, oxidative stress

## Abstract

**Objectives:**

Synthetic biology has emerged from molecular biology and engineering approaches and aims to develop novel, biologically-inspired systems for industrial and basic research applications ranging from biocomputing to drug production. Surprisingly, redoxin (thioredoxin, glutaredoxin, peroxiredoxin) and other thiol-based redox systems have not been widely utilized in many of these synthetic biology applications.

**Methods:**

We reviewed thiol-based redox systems and the development of synthetic biology applications that have used thiol-dependent parts.

**Results:**

The development of circuits to facilitate cytoplasmic disulfide bonding, biocomputing and the treatment of intestinal bowel disease are amongst the applications that have used thiol-based parts. We propose that genetically encoded redox sensors, thiol-based biomaterials and intracellular hydrogen peroxide generators may also be valuable components for synthetic biology applications.

**Discussion:**

Thiol-based systems play multiple roles in cellular redox metabolism, antioxidant defense and signaling and could therefore offer a vast and diverse portfolio of components, parts and devices for synthetic biology applications. However, factors limiting the adoption of redoxin systems for synthetic biology applications include the orthogonality of thiol-based components, limitations in the methods to characterize thiol-based systems and an incomplete understanding of the design principles of these systems.

## Introduction

**Figure 1.**
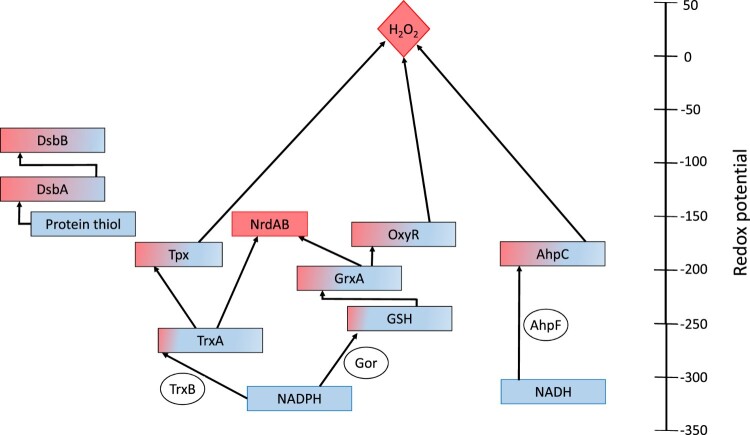
A sample of the *Escherichia coli* thiol-redox network showing the kinetic and spatial separation of electron flow pathways. Within the cytoplasm, reducing equivalents from NADPH and NADH are used by the reductases (circles), thioredoxin reductase (TrxB), glutathione reductase (Gor) and alkyl hydroperoxide reductase subunit F (AhpF) to reduce thioredoxin (Trx), oxidized glutathione and alkyl hydroperoxidase subunit C (AhpC) respectively. In turn, other targets such as glutaredoxins (Grx), ribonucleotide reductase (Nrd), thiol-peroxidase (Tpx) and the transcription factor OxyR, are reduced by thiol-disulfide exchange [6,7]. Within the periplasmic space, protein thiols are oxidized by DsbA which in turn is oxidized by DsbB [8]. Arrows show the electron flow pathways between cognate redox partners. The redox potentials for GrxA, TrxA and DsbA were obtained from Ref [9]; the redox potentials for *E. coli* AhpC and Tpx were assumed to be similar to Salmonella typhimurium AhpC [10], while the redox potential for DsbB was also assumed to be a midway between the isolated DsbB and ubiquinone [11]. The hypothetical distribution between the oxidized (pink) and reduced (blue) moieties are shown for each redox couple with the NADPH and NADH electron sources shown in blue and, the hydrogen peroxide and ribonucleotide reductase electron sinks shown in pink.

Thiol-based systems, such as redoxins, have been found in all living organisms where they play critical and wide-ranging roles in intracellular redox regulation, antioxidant defense, DNA synthesis and sulfur metabolism amongst other functions [1–6]. Redoxin proteins utilize thiol-based chemistries to reduce or oxidize their target molecules, and their activities are restored by coupling to other redox partners forming systems of reactions. While some redoxin systems share common redox partners or targets, they often function as discrete systems based on their intracellular location and the kinetic affinities for their cognate redox partners (Figure 1). Consequently, redoxin systems can be involved in distinct physiological roles *in vivo* [12–16].

Dysregulation of redoxin activity has been associated with a number of non-communicable diseases [17–21], and in pathogens, thiol-based systems play critical roles in their survival [1,22–24]. In addition to redoxins, an ever-expanding repertoire of proteins also appears to be regulated by thiol-based mechanisms, including phosphokinases [25–30], transcription factors [31–35], proteases [36,37] and structural proteins [38–40]. However, despite their ubiquitous nature and the multifunctional roles of redoxin systems, redoxin components have not been widely used for synthetic biology applications. In the sections that follow, we consider how synthetic biology circuits are developed, circuits that have utilized thiol-based components, and thiol proteins that could be purposed for synthetic biology applications. We also consider challenges for the development of redox synthetic biology with a focus on microbial synthetic biology circuits, where many of the design principles for synthetic biology applications were developed.

## Building synthetic biology circuits

Synthetic biology has been defined as ‘the engineering of biology: the synthesis of complex, biologically-based (or inspired) systems, which display functions that do not exist in nature’ [41] and its emergence has led to a wide range of applications ranging from genetic circuits for monitoring metabolites, engineered foods, cellular computing and many other applications [42–48]. Consensus on this definition is not universal as it could be argued that the development of synthetic cells [49,50], genome-wide editing approaches [51], novel DNA base-pairs [52] and genome-wide engineering efforts [53] are also examples of synthetic biology approaches. Further, as synthetic biology borrows heavily from engineering and, molecular and systems biology approaches, the demarcations between these fields are ambiguous. Nonetheless, perhaps a characteristic feature of synthetic biology is the emphasis on higher-order abstraction in which distinct ‘parts’ are combined into ‘modules’ and then into ‘circuits’ [42]. We will use the definition of a biological ‘component’ as a protein, protein domain, or nucleic acid sequence and a ‘part’ represents a process or system that performs a particular function. A module represents a functional combination of parts with defined inputs and outputs, while a circuit represents the entire system of parts and modules. Within this framework, biological parts are the key functional units for building circuits [42]. A significant aim for synthetic biology is the development of circuits *de novo* using a range of standardized, interoperable parts, which has led to the development of parts lists [54] such as the International Genetically Engineered Machine (iGEM) competition repository (http://parts.igem.org/Main_Page) [55].

The development of biological circuits can be traced to the description of the *lac* operon by Jacob and Monod [56]. This pioneering work inspired recombinant cloning and expression technologies that used relatively simple switches for artificial gene regulation. With the recognition that even complex biological networks could be de-convoluted into simpler modules, genetic circuits such as toggle switches [57] and repressilators [58] were developed in the early 2000s. These circuits comprised parts that recognized environmental inputs, effected a logical operation using transcriptional components and fed the results of this operation to a GFP-reporter to reveal these behaviors. These initial studies have since been followed by a slew of transcriptional and translation circuit designs such as RNA-based systems, quorum-sensing and sensory circuits [54,56,59–62].

Non-transcriptional synthetic biology circuits based on metabolic and signaling pathways have also been developed, such as the artemisinic acid pathway in yeast [63] and methods to reprogram the flux in *Escherichia coli* [64]. Of particular interest to this review is the development of synthetic phosphokinase signaling circuits, which involve modifying the cellular signaling proteins or adaptor proteins to generate novel behaviors [65]. Interestingly, these studies have not only led to the design of novel synthetic circuits but have also revealed the design principles of natural signaling systems [66,67]. For example, studies using synthetic circuits have revealed that phosphokinase signaling systems are modular, consisting of distinct sensor (input), information processing and response (output) layers that operate at different timescales [65]. In these systems, inputs are rapidly processed through the sensor and information kinetic processing layers, while the outputs of these systems, such as transcriptional regulation or cell fate decisions, typically occur at longer timescales. Remarkably, the functional separation into input and output functions can sometimes be found in individual signaling proteins, which contain distinct signal recognition, transmission and effector domains [65]. The modular nature of phosphokinase signaling systems and signaling proteins has led to several interesting applications, such as immune cell hacking [68] and the generation of novel circuits using chimeric signaling proteins [65,69].

Despite the many successes, there have been some challenges with developing synthetic circuits, with the most significant of these being orthogonality. Ideally, synthetic biology parts, components and circuits should be orthogonal (independent) of their host cell and show predictable, context-independent behavior. However, this is often not the case as synthetic systems inevitably influence, and are influenced, by the cellular environment they are embedded within [70]. The characterization and performance of synthetic biology components and parts therefore remains a limiting factor for synthetic biology applications [54,61]. Accordingly, an iterative rational design strategy has been used for building these circuits: computational/mathematical modeling, circuit construction and experimental analyses followed by multiple iterations to obtain a desired phenotype [42,54]. This strategy remains the workflow for most synthetic biology studies, although other strategies to develop biological circuits include combinatorial selection or directed evolution [61]. Notably, the methodologies described in this design strategy have already been used for thiol-based systems.

## Thiol-based redoxins and redoxin systems

The structural, biochemical and kinetic properties of redoxin components have been extensively reviewed [4,12,71,72], and using *E. coli* as a model, we will focus on the basic features of these systems. Many cytoplasmic redoxin systems show a similar configuration: an external source for reducing equivalents, usually NAD(P)H; pyridine nucleotide-disulfide oxidoreductases that convert these equivalents into thiol redox power and thiol-disulfide exchange hubs that then transfer electrons to redox partners and oxidant sinks. The reactions within these hubs are responsible for the functions most associated with redoxin systems, including metabolism, antioxidant defense, redox regulation, DNA synthesis and signaling.

### Reducing sources

The reducing power for many of these systems, NADPH, is generated from the pentose phosphate pathway and depending on the cell type, the oxidation of isocitrate, malate or methylene tetrahydrofolate [2,73]. In addition to its role in antioxidant defense, NADPH provides the reducing power for several cellular anabolic reactions [74,75] and is also involved in the generation of reactive oxygen species (ROS) by NADPH oxidases [76]. However, in some bacteria, NADH provides reducing equivalents for alkyl hydroperoxidases (Figure 1). NAD^+^ is an electron acceptor in many catabolic reactions [77] and in most cell types, the NAD^+^:NADH ratio favors the oxidized isoform (see for example [78,79]). It is unclear whether NADH pools are limiting for these redoxin systems. In specialized compartments such as the periplasm or endoplasmic reticulum, polypeptide thiol groups which become oxidized and isomerized into native disulfide bonds, are the reducing source for these pathways [80,81]. The reducing power for redoxin systems in plant chloroplasts, is obtained from the thylakoid electron transport chain. Here, electrons obtained following light harvesting are used to reduce ferredoxin, which reduces ferredoxin-thioredoxin-reductase, which in turn, reduces plant thioredoxins [82,83].

### Reductases

Reducing equivalents from NAD(P)H are transferred to oxidized thiol redox couples by members of the pyridine nucleotide-disulfide oxidoreductase family [2,84]. These reductases exist as homodimeric proteins, with each monomer containing a cofactor binding domain, a flavin adenine dinucleotide (FAD) group and a thiol-disulfide redox active site. Upon binding of the NAD(P)H cofactor, electrons are shuttled to the FAD domain and then to the thiol-disulfide redox domain [1,85]. Interestingly, the flow of electrons within these proteins from the NAD(P)H to the disulfide active site domain mirrors the overall configuration of redoxin systems (Figure 1).

Thioredoxin reductases are found in two classes, high and low-molecular weight, with the high molecular weight thioredoxin reductase being closely related to glutathione reductase [2,86]. The high molecular weight thioredoxin reductase, which is largely found in metazoans, uses a Cys-selenocysteine pair to reduce thioredoxin and displays a broader substrate specificity than the lower molecular weight thioredoxin reductase which employs a Cys-Cys pair for disulfide reduction [2,86]. In *E. coli,* loss of thioredoxin reductase converted thioredoxins into oxidases, showing that these systems’ coupling and connectivity is key to their activity [87]. Glutathione reductases all share a common ancestor and reduce oxidized glutathione (GSSG) to two individual glutathione (GSH) moieties [1,2,86,88]. Depending on the organism, other reductases such as alkyl hydroperoxide reductase subunit F, coenzyme A disulfide reductase, dihydrolipoamide reductase, coenzyme A-glutathione reductase or trypanothione reductase may supply reducing equivalents to components within the thiol-disulfide hub [2].

In *E. coli* a functional thioredoxin or glutaredoxin system is usually required to support ribonucleotide reduction under aerobic conditions [89,90]. However, genetic screens have revealed that loss of both thioredoxin and glutaredoxin reductase activities in *E. coli* (Δ*trxBΔgor*) cells was rescued by mutations that introduced a single amino residue into the sequence of the AhpC peroxidase allowing it to support the reduction by the glutathione/glutaredoxin pathway [91,92]. Similarly, in *E. coli* Δ*trxBΔgshA* mutants, loss of thioredoxin reductase and GSH biosynthesis activity was also compensated by a mutant AhpC, suggesting that this enzyme shows a high degree of functional plasticity [93], which has been exploited in some synthetic biology applications (below).

### Thiol-disulfide exchange hub

The thiol-disulfide exchange hub is a central hub of the cellular redox network [94,95], and its constituents depend on the organism and its subcellular location. Within the cytoplasm, this hub reduces a large range of targets by thiol-disulfide exchange (Figure 1). These targets are involved in both metabolic (bulk flow) and signaling (state changes) reactions and, depending on their connectivity, can show emergent behaviors such as ultrasensitivity [96]. The components within this hub may therefore be useful for synthetic biology applications.

Thioredoxin is an ancient [97,98] ubiquitously distributed protein that reduces disulfide substrates under normoxic conditions by virtue of its redox potential and coupling to NADPH via thioredoxin reductase. This redoxin plays essential roles in DNA synthesis, redox regulation and antioxidant defense [1,12]. In this latter role, thioredoxin provides reducing equivalents for thiol peroxidases and modulates signaling pathways [1,99,100]. In plants, thioredoxin plays a critical role in the light-dependent regulation of the chloroplast Calvin-Benson cycle [40,82,83].

Analyses of thioredoxin protein sequences and structures have revealed a conserved -WCXXC- catalytic amino acid motif and a signature thioredoxin fold consisting of a five-stranded β-sheet surrounded by four α-helices [101,102]. The active-site catalytic cysteine is present on a loop of one of the four α-helices, allowing it to readily participate in nucleophilic attacks on disulfide bonds. The second Cys resolves the resulting mixed disulfide in the thioredoxin active site leading to oxidized thioredoxin [103,104]. While thioredoxin’s structural characterization has been well described, the kinetic characterization of this redoxin and indeed other redoxins have had challenges. Redoxins constitute a moiety conserved couple [105,106] in which the total sum of the redoxin pool does not change but instead distributes between the reduced and oxidized isoforms [107,108]. As these reduced and oxidized isoforms can be quantified *in vivo,* studies have correlated the thioredoxin redox potential with physiological conditions associated with oxidative stress [109–112]. However, it has been argued that redox potentials are inaccurate measures because cellular systems exist far from equilibrium [113–115]. Thioredoxins were also considered enzymes and were consequently characterized by Michaelis–Menten parameters and fluxes *in vitro* [116]. The relationship between these distinct *in vitro* and *in vivo* measures had been obscure but recent work by our group has shown that the redox potential and a new measure, the thioredoxin redox charge (reduced thioredoxin/total thioredoxin), are in fact linearly correlated to the flux by the flux-force relationship [117]. Thus, observed changes to the thioredoxin redox charge *in vivo* reflect the total demand for reducing power from the thioredoxin system.

Most organisms also possess a low-molecular weight thiol pool which is present at high (millimolar) concentrations. GSH fulfills this function in many cells [118] (Figure 1), although other low-molecular weight thiols such as co-enzyme A, gamma-glutamyl cysteine, mycothiol, trypanothione, bacillithiol, ergothioneine and ovothiol appear to perform this role in other cells [119–124]. These thiols provide a source of reducing power for peroxidase, disulfide and mixed-disulfide reactions [125] and, act as general redox ‘buffers’ that protect against ROS [126]. This latter role was disputed because GSH has a relatively low rate constant with hydrogen peroxide compared to specialist peroxidases [125,127]. However, GSH may nonetheless be effective against diffusion-limited species such as the hydroxyl radical because of its high intracellular concentration.

GSH plays an additional antioxidant role. Glutathionylation, which is the addition of glutathione to labile cysteine residues, protects these residues from ROS oxidation and can regulate protein function under both oxidative stress and normoxic conditions [3]. Glutaredoxins play a central role in the glutathionylation/deglutathionylation cycle and it was initially believed that there were two distinct pathways for reducing disulfides and mixed disulfide in dithiol glutaredoxins viz. the mono – and dithiol mechanisms [128]. Although apparently distinct, it was shown that the glutaredoxins utilize either mechanism [129,130], with the dithiol mechanism probably required to resolve glutaredoxin mixed disulfides [131]. Other systems that use low-molecular weight thiols, other than GSH, often have cognate redoxins to reduce mixed disulfides (e.g.) mycoredoxins reduce mycothiolated proteins in *Mycobacterium tuberculosis* (see for example [132]). Note that monothiol glutaredoxins do play critical roles in metal ion coordination and excellent reviews on these proteins are available [132,133].

### Hydrogen peroxide detoxification and detection

Increases in oxygen, approximately two billion years ago, led to ROS generation as an inadvertent by-product of oxygen metabolism. These reactive species can damage crucial cellular components such as nucleic acids, proteins and lipids [103,134]. The generation of ROS led to the evolution of molecular antioxidant machinery, tasked with protecting cells from critical component damage and enabled both ROS-signaling and regulatory pathways to emerge [103,135]. Hydrogen peroxide, in particular, appears to play an essential role in cell signaling [16,125] and conveniently can serve as an input for synthetic biology applications (below). The primary receivers for these inputs are peroxiredoxins and redox-sensitive transcription factors.

Peroxiredoxins are amongst the most abundant in proteins in cells [13,136–138]. Like thioredoxins, peroxiredoxins are ubiquitously distributed through all living kingdoms and consist of six evolutionary subfamilies: Prx1, Prx5, Prx6, Tpx, PrxQ and AhpE [139,140]. Peroxiredoxins have also been classified as 2-Cys typical, 2-Cys atypical and 1-Cys peroxiredoxins according to the number and position of their active-site conserved cysteine residues and their ability to form intra- or intermolecular disulfide bonds [126–128]. Oxidation of dimeric, typical 2-Cys peroxiredoxins, results in an intermolecular disulfide formation between the peroxidatic (Cp) and resolving (Cr) active site cysteines of the partner subunits while in atypical 2-Cys peroxiredoxins, oxidation results in an intramolecular disulfide bond [141–143]. 1-Cys peroxiredoxins are usually monomers, and oxidation leads to a mixed disulfide formation with the low-molecular weight thiol system, which can be reduced by its cognate redoxin [144,145].

Peroxiredoxins react rapidly with hydrogen peroxide (10^4^–10^8^ M^−1^·s^−1^) using a reaction mechanism that results in the formation of a sulfenic acid (SOH) on the peroxidatic cysteine [13–15,125]. This sulfenic acid can condense to a disulfide bond [146,147] or form a mixed disulfide with a low-molecular weight thiol such as GSH [148]. Interestingly, disulfide bond formation is significantly slower in some eukaryotic peroxiredoxins when compared to their prokaryotic homologs [136,149]. Here, the peroxidatic cysteine can be reversibly hyperoxidized to a sulfinic (SO_2_H) [137] or irreversibly oxidized to a sulfonic (SO_3_H) acid derivative [14,150,151]. This mechanism preserves the reduced thioredoxin pool [139], may facilitate oxidation of phosphokinase signaling proteins (‘floodgate’ hypothesis [150]), and also leads to the assembly of hyperoxidized peroxiredoxins into dodecameric molecular chaperones [136,147]. In mammalian cells, hydrogen peroxide-induced inactivation of protein tyrosine phosphatases are important for signal propagation through phosphokinase cascades [152]. However, it has been unclear how hydrogen peroxide could inactivate these phosphatases in the presence of peroxiredoxins. Recent work has shown that peroxymonocarbonate, formed by the reaction between hydrogen peroxide and bicarbonate, can facilitate signaling by oxidizing protein tyrosine phosphatases and hyperoxidizing peroxiredoxins [153–155].

Peroxiredoxins are also mediators for sensor-mediated transcription redox signaling [13,15,127,141,151,156]. An example of this type of transcriptional regulation is the activation of the Yap1 transcription factor by glutathione peroxidase 3 (Gpx3/Orp1) in *Saccharomyces cerevisiae* [157]. Following the oxidation of the Gpx3 peroxidatic cysteine to a sulfenic acid by hydrogen peroxide, this peroxiredoxin can together with Ybp1, oxidize the C-terminal cysteine residues in Yap1 [158]. Subsequent oxidation events lead to the formation of 2–3 disulfide bridges in Yap1, which mask its nuclear export signal allowing it to localize in the nucleus and activate gene transcription [159,160]. Similar sulfenic acid-dependent mechanisms are used by many other redox signaling systems [4,16,127,143] and thus, peroxiredoxin hyperoxidation (and recovery) may simply be an unavoidable consequence of this type of signaling chemistry.

Bacterial cells are exquisitely sensitive to hydrogen peroxide and other oxidative stressors [34,35]. The transcription factor OxyR is found in Gram-positive and some Gram-negative bacteria and is directly oxidized by hydrogen peroxide and other oxidants [32,33,161]. In *E. coli*, exposure to hydrogen peroxide results in OxyR Cys199 becoming oxidized to a sulfenic acid which condenses into a disulfide bond with Cys208 [32,162]. Formation of this bond triggers a conformational change to the transcription factor that allows it to induce the transcription of the antioxidant genes in the OxyR regulon [163]. In *E. coli*, the OxyR reaction with hydrogen peroxide is very rapid and shows a strong cooperative character with an estimated *in vivo* Hill coefficient of 10 [156,164]. While thioredoxin can reduce OxyR *in vitro,* the transcription factor is primarily reduced by the glutaredoxin system *in vivo,* highlighting the kinetic specificity of these systems ([32,162], cf. Figure 1).

## Areas of application for thiol-based components and systems in synthetic biology

Thiol-based components and systems have been utilized in a number of applications ranging from circuits to biosensors (Table 1) which are discussed in detail below.

**Table 1.** Examples of synthetic biology applications that utilize thiol-based components

Application	Components
Biocomputing	OxyR
Inflammatory bowel disease	OxyR
Cytoplasmic disulfide bond formation	AhpC*, GrxA, PDI-Grx, DsbC, GSH
ROS sensing	cpYFP, roGFP, rxYFP
Engineered enzymes	NAD(P)H reductases
Biomembranes	4-arm thiolated polyethylene glycol, lysozyme
Intracellular peroxide generators	D-amino oxidases, KillerRed

### Circuits

**Figure 2.**
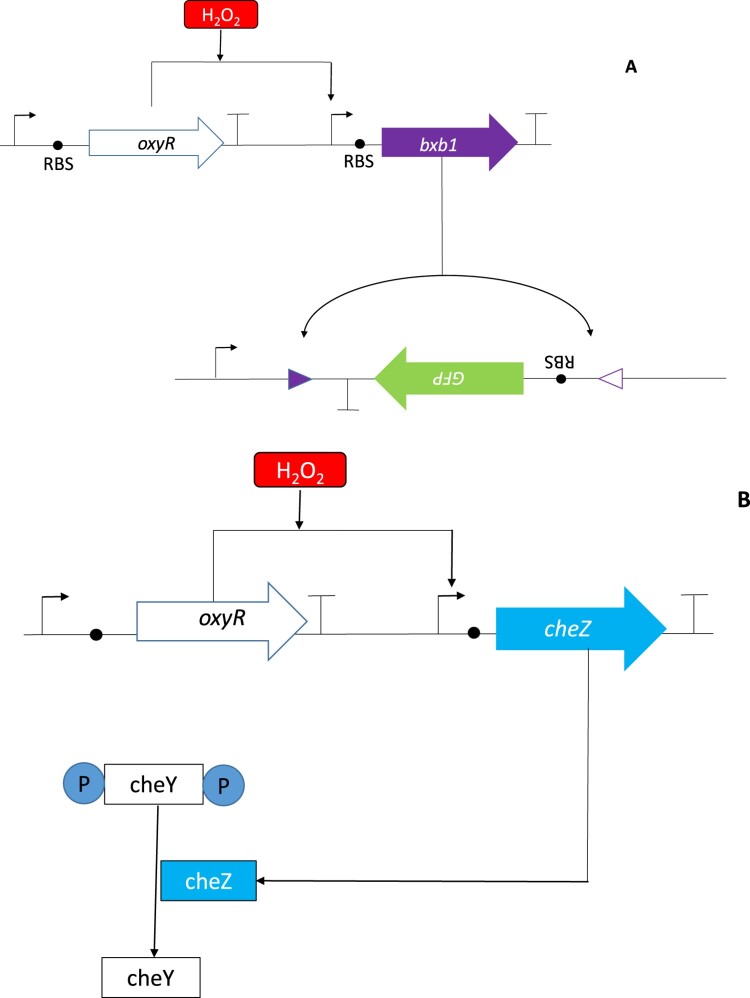
OxyR is used as a specific hydrogen peroxide sensor in genetic circuits (A) Genetic comparator circuit built using an OxyR part. In this circuit, OxyR activation by hydrogen peroxide results in the expression of the recombinase Bxb1 which recognizes a computationally designed ribosome binding site (RBS). Recombination of these sites leads to GFP expression [165]. By adjusting the ribosome binding sequences, promoter (↱) and terminator (Т) sequences, the GFP-output of this part could be varied. In (B), OxyR was used to induce the transcription of the CheZ phosphatase which dephosphorylated CheY which subsequently reduced *E. coli* tumbling [166].

Only three synthetic biology circuits have been developed using thiol-based components to the best of our knowledge. In the first of these circuits, OxyR was expressed on a low copy number plasmid and in response to hydrogen peroxide, induced the expression of a Bxb1 recombinase (Figure 2A). Once expressed, the Bxb1 recombinase excised, flipped and recombined a GFP-reporter sequence which was expressed from a bacterial artificial chromosome. Interestingly, a hyperbolic GFP expression profile in response to hydrogen peroxide was observed in a synthetic circuit that matched the cooperative activation of OxyR. By adjusting the sequences of the ribosomal binding and transcriptional activator sites, this part’s performance was modified to create comparators, band-pass filters, analogue-to-digital converters and mixed signal gene circuits for bio-computation [165].

Redox synthetic biology applications could play important roles in elucidating the etiology and treatment of both chronic and acute diseases that involve redox dysregulation. Inflammatory bowel disease (IBD) and other gastrointestinal pathologies are associated with oxidative stress [167,168] and a circuit was developed which allowed engineered *E. coli* to migrate towards hydrogen peroxide [166]. The swimming motion of *E. coli* is controlled by the rotation of its flagella motors. If the motors run counter-clockwise, the flagella rotate as a bundle resulting in a ‘running’ motion associated with chemotaxis. However, if any motor rotates clockwise, the bundle breaks up and the cells’ tumble’. This clockwise rotation of the motor is controlled by CheY which binds to the motor when phosphorylated while its dephosphorylation is effected by the CheZ phosphatase [169].

The control of this naturally occurring system was engineered to be sensitive to hydrogen peroxide (Figure 2B). In this circuit, oxidation of OxyR by hydrogen peroxide-induced the transcription of the phosphatase CheZ which therefore allowed *E. coli* to migrate towards hydrogen peroxide. While hydrogen peroxide at high concentrations can be toxic, it is envisioned that such engineered probiotic cells could ‘pseudotax’ towards regions in the gut with oxidative stress where they could detoxify hydrogen peroxide and reduce the ROS burden within the host gut [166].

While oxidative stress can be detrimental to cells, hypoxia also plays a central role in diseases including ischemic heart disease and cancer and, is associated with both immune system activation and inflammation [170]. Recently, it was shown that hypoxia-inducible-factor (HIF-1), the master regulator of the hypoxic response in mammalian cells, was regulated by protein disulfide isomerase [171]. Given the advances in delivery systems ranging from polymers [172], viral vectors [173] and biohybrid bacteria [174], we anticipate the development of thiol-based redox circuits that are activated in response to hypoxic conditions.

Arguably, the most comprehensive redox synthetic biology circuits are those that aimed to facilitate cytoplasmic disulfide bond formation in *E. coli* which led to the Origami (Novagen), SHuffle (NEB) [175] and SHuffle2 strains [176]. In these cells, the functional separation and redundancy between the NADPH and NADH-dependent thiol-based reactions (cf. Figure 1) was exploited. Origami strains contained thioredoxin reductase (*trxB*) and glutathione reductase (*gor*) mutations that supported cytoplasmic disulfide oxidation. These cells also possessed a modified NADH-depedent AhpC protein (AhpC*) to reduce glutaredoxin (GrxA) and support cell growth [91,92,177].

**Figure 3.**
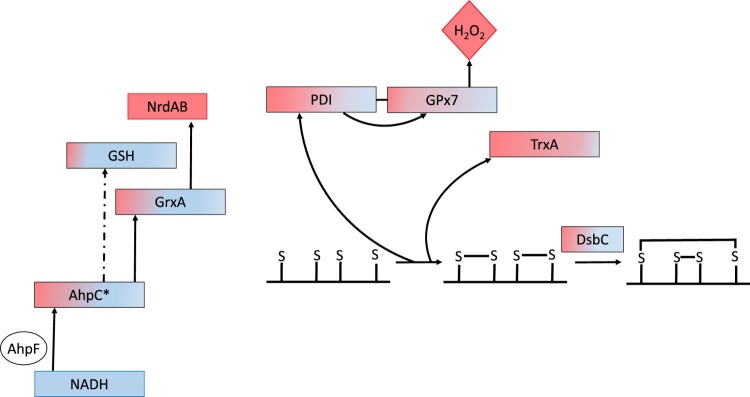
Electron flow pathways within *E. coli* SHuffle2 cells that support cytoplasmic disulfide bond formation. In these cells, thioredoxin and glutathione reductase have been deleted (cf. Figure 1), and a mutant peroxidase (AhpC*) reduced glutathionylated glutaredoxin and GSH [92] to support metabolic pathways such as ribonucleotide reductase (Nrd) cycling. A PDI-GPx7 chimera was used to reduce hydrogen peroxide and, together with thioredoxin, oxidize protein thiols, while disulfide isomerization by DsbC was used to enable correctly folding of a target antibody [176]. The hypothetical distribution between the oxidized (pink) and reduced (blue) moieties are shown for each redox couple.

In SHuffle cells, the periplasmic isomerase DsbC was cytoplasmically expressed to facilitate the correct folding of target proteins although efficient folding of a range of proteins depended on strain backgrounds, expression parameters and helper proteins [175]. Using redox-sensitive probes and transcriptional analysis, it was shown that SHuffle cells experienced hydrogen-peroxide stress presumably because their thioredoxin and glutathione pathways had been disrupted which impacted recombinant protein expression [178]. This problem was ingeniously solved by coupling human protein disulfide isomerase to the thiol peroxidase GPx7, creating a redox cascade in which oxidizing equivalents were transmitted from hydrogen peroxide to target proteins (Figure 3). In both strains, efficient folding and production of a range of proteins depended on strain backgrounds, expression parameters and helper proteins, highlighting the complexity of protein folding [170,171]. In addition, these results could also suggest that the construction of synthetic redoxin circuits may also face an orthogonal problem.

### Molecular sensors

**Figure 4.**
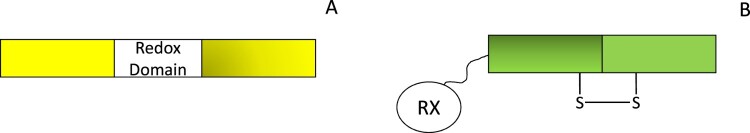
Genetically encoded redox sensors have been developed using circularly permuted fluorescent proteins (A) or redox-sensitive fluorescent proteins (B). Circularly permuted redox proteins contain a redox domain that can bind specific redox species which perturbs their fluorescent output. In contrast, redox-active fluorescent proteins contain a flexible linker region on either the C- or N- terminus and, a redoxin protein (RX) that transfers redox equivalents to redox-sensitive cysteines on the fluorescent protein, affecting probe fluorescence.

Fluorescent proteins have been used as outputs in synthetic circuits and have been used to develop genetically encoded fluorescent redox sensors to measure different redox species in specific intracellular locations [179–181]. These sensors are likely to be important components for redox synthetic biology applications and can be divided into two broad categories: circularly permuted redox sensors and redox-active fluorescent proteins (Figure 4). Circularly permuted fluorescent proteins were developed by swapping the amino- and carboxyl parts of fluorescent proteins and introducing a linker region between these regions [182,183]. Changes to the linker induced by binding to a specific ligand affects the circularly permuted protein structure and, consequently, its fluorescent signal [180,181]. The hydrogen peroxide probe, HyPer, was created by fusing the OxyR regulatory domain into the linker region of circularly permuted yellow fluorescent protein (cpYFP). Oxidation of the cysteines within this linker region induced a conformational change that changed cpYFP’s ionization state and fluorescence [184]. Several redox sensors have since been developed using this strategy resulting in a range of circularly permuted sensors that detect different redox species [181,185].

Redox-active fluorescent proteins, on the other hand, have been generated by introducing redox cysteine residues into fluorescent proteins to form a redox-sensitive yellow fluorescent protein (rxYFP) and a redox-sensitive green fluorescent protein (roGFP) [179,186–188]. Oxidation of these cysteines modifies the structure of these proteins and the fluorescent output of the sensor. While redox species can directly oxidize the cysteine residues within rxYFP and roGFP, these reactions are significantly faster in the presence of redoxin proteins. Therefore, redox sensors have been developed with rxYFP or roGFP connected by amino or carboxyl Ser-Gly linker regions to redoxin proteins (e.g.) Grx1-roGFP2 is sensitive to the GSH/GSSG ratio [186,189–191].

Some properties of these fluorescent proteins are important considerations for their use in synthetic biology applications. First, some of the earlier probes were pH-sensitive and therefore, their outputs did not necessarily reflect genuine redox-dependent changes [179]. Second, many of these sensors’ dynamic ranges can be quite different, and some probe signals are ratiometric to avoid photobleaching effects [179,180]. A final consideration is that some of these probes are extremely sensitive and therefore, care must be exercised to avoid artefacts that could arise from media components (see for example [192]). Nonetheless, these sensors show that in principle, different redox sensitive domains can be swapped between proteins to create chimeric redox proteins with novel behaviors.

### Engineered enzymes

NAD(P)H provides reducing power for most thiol-based redox systems, and in response to oxidative stress, the flux through the pentose phosphate pathway increases to support thiol-dependent antioxidant defenses. It was initially reasoned that this resulted from glyceraldehyde 3-phosphate dehydrogenase oxidation and consequent rerouting of the flux. However, it has since been shown that the pentose phosphate pathway contains excess capacity because the limiting enzyme in this pathway, glucose-6-phosphate dehydrogenase, shows strong inhibition by NADPH [193,194]. Thus, increased demand for NADPH can be matched by an increase in the flux of this pathway.

Nonetheless, we foresee that synthetic parts based on redoxin systems may require a higher NADPH demand. There have been attempts to engineer NADPH-dependent dehydrogenases to oxidize NADPH more efficiently, particularly for industrial applications. Zhang et al. [195] developed an *in vivo* platform to discover such enzymes by engineering *E. coli* glycolysis to utilize the NADP^+^/NADPH couple rather than NAD^+^/NADH couple under anaerobic conditions. To maintain redox balance and cell growth, oxidation of NADPH by a D-lactate dehydrogenase from *Lactobacillus delbrueckii*, allowed for the selection of mutant oxidoreductase enzymes with improved kinetic efficiency [195]. It would be interesting to determine if such a selection platform could also be developed to select more efficient oxidoreductases for redoxin systems.

Site-directed mutagenesis studies of redoxins have elucidated many of the structure/function relationships of redoxins. For example, in *E. coli* Δ*dsbA* mutants, thioredoxin could act as an oxidant when exported to the periplasm. Strikingly, swapping this thioredoxin’s Cys-Gly-Pro-Cys active site with a DsbA Cys-Pro-His-Cys active site motif led to oxidation kinetics that were equivalent to wild-type (DsbA^+^) strains [196]. These and other studies led to the concept of the active site CXXC motif acting as a rheostat with changes in the amino acid sequence motif changing the redox potential of the redoxin [197]. While changes in this sequence also affected these enzymes’ catalytic activities [198], the overall thioredoxin fold was still a significant contributor to redox potential [199]. Similarly, studies with glutaredoxins and peroxiredoxins have shown precisely how their activities can be modified by site-specific changes (see for example [200,201]). Collectively, these studies highlight how redoxins may be modifiable for synthetic biology applications.

### Biomaterials

Thiol-disulfide chemistries are used for the formation of many membranes and films [202]. For example, mucin glycoproteins are critical components in hydrogels that play important roles in protecting the gastrointestinal, urinary and respiratory tracts [203]. Dimerization of the mucin-2-glycoprotein depends on the formation of disulfide bonds [204]. Analogously, synthetic hydrogels are biopolymers with utility in several applications such as drug delivery, cell-free synthesis and tissue engineering. One method to produce these polymers is to use a thiolated-polymer, such as 4-arm thiolated polyethylene glycol, which can be cross-linked by disulfide bonds into a gel. Horseradish peroxidase is often used to catalyze the reaction, which can be further enhanced with the addition of phenolic compounds. The resulting gels can encapsulate cells, be used for cell-free protein synthesis and deliver proteins or drugs [205]. Significantly, these hydrogels can be dissolved by reduction with low-molecular weight thiols and could therefore be used in controlled release applications. In a different approach, a protein-based nanofilm was developed by reducing the Cys6-Cys27 disulfide bond in lysozyme which could then oxidize and aggregate to form a proteinaceous film that encapsulated a range of molecules and particles [206].

### Intracellular hydrogen peroxide generators

Given that hydrogen peroxide is already used as an input for redox synthetic biology circuits, the controlled generation of intracellular hydrogen peroxide could also be an important tool for synthetic biology applications and two approaches may be relevant here. First, D-amino oxidases catalyze the deamination of D-amino acids to their corresponding imino isoforms with concomitant hydrogen peroxide production. Because most cellular amino acids are in the L-stereoisoform, the enzyme will only be active once a D-amino acid, such as D-alanine, is introduced into the media. Further, specificity can also be ensured by targeting the enzyme to specific cellular compartments [207,208]. The second approach uses the genetically encoded photosensitizer, KillerRed (KR), which generates ROS in the presence of green light in particular [209]. By fusing KR to SOD1, Laporte *et al.* were able to generate hydrogen peroxide on demand in insulin cells [210]. We foresee that both these approaches may be useful in activating synthetic biology circuits.

## Challenges and questions for building a thiol-based synthetic biology ecosystem

Thiol-based redox systems appear to have all the ingredients for their adoption in synthetic biology. Redoxin protein domains can be combined in a modular fashion to generate novel functions and, the development of cells specializing in cytoplasmic disulfide bond formation shows that synthetic redox circuits may also have commercial value. Moreover, the first step in synthetic biology applications is computational modeling and several models of thiol-based systems are already available ([115], Table 2). These models can presumably be adapted for synthetic biology applications, although surprisingly, models of thiol-oxidation pathways have not yet been developed. We highlight three questions and challenges that must be addressed before thiol-based systems are used more extensively in synthetic biology applications.

**Table 2.** Computational models of redoxin systems.

Cell type	Compartment	Type	Reference
Jurkat T-cell	Cytoplasm	ODE	[211]
*Escherichia coli*	Cytoplasm	ODE	[96]
Mammalian	Cytoplasm	ODE	[212]
HeLa	Cytoplasm	ODE	[213]
Human erythrocyte	Cytoplasm	ODE	[214]
Fission yeast	Cytoplasm	ODE	[215]
HeLa	Mitochondria	ODE	[216]
Head and neck cancer	Multi	Flux-Balance	[217]

### Are the design principles of thiol-based systems understood?

In some cases, such as the development of Origami and SHuffle cells, the design principles of thiol-based systems were sufficiently understood to facilitate these applications. In particular, this work showed that the redundancy within redoxin networks could be exploited to allow for certain systems to be modified for a novel function while its counterpart system is used to support thiol-dependent cell metabolism. However, it is less clear whether our understanding of other thiol-based systems is sufficient to rationally develop other synthetic biology applications (e.g.) what are the advantages of using OxyR compared to the Yap1 as a hydrogen peroxide detector? It is clear that further theoretical and experimental efforts are needed to elucidate the design principles of these systems to accelerate their adoption into synthetic biology applications. We also anticipate that the development of redox synthetic circuits, in turn, will uncover additional design principles of these systems.

### Can thiol-based systems overcome the orthogonal problem in synthetic biology?

Given that redoxin proteins are universally distributed and are ‘moonlighting’ proteins with broad substrate specificities [218–220], it is likely that redoxin parts and circuits will interact with their host cells. However, the kinetic affinities between many redoxin components have been determined which could, in principle, allow for a choice of components that confer some specificity to parts (Figure 1). Further, as shown for roGFP probes, specificity could also be achieved by tethering synthetic redoxin components to particular redoxins.

### How should thiol-based synthetic biology parts be characterized?

Many redoxin systems are uncharacterized or partially characterized and the kinetic parameters for many redoxin components, even in well-defined systems, are not readily available [115]. It could be argued that the higher-level abstraction and iterative refinement strategy used in synthetic biology allows for part testing until a specified objective is reached and therefore, the kinetic details of every component are not necessary. However, as computational modeling is often the first step in developing synthetic biology circuits, this limitation can reduce this design strategy’s effectiveness. Further, the connectivity within many redoxin systems, particularly from organisms inhabiting niche redox microenvironments, can also be significantly different from model organisms. This suggests that a wide variety of novel parts could be constructed using these redoxins and there is an urgent need to develop methods to rapidly characterize and compare these systems. The development of redox sensors and analytical methods to determine the flux [117] offers a potential solution to this problem.

## Conclusion

Redoxin systems could offer new components and parts for synthetic circuits and synthetic biology applications. On the other hand, synthetic biology approaches could offer insights into the design principles of thiol-based systems that could improve our understanding of these systems and their dysregulation during disease. To bridge these fields, there is a need to develop better and preferably, high throughput methods to characterize redoxin systems and understand how they function as parts. These efforts would complement existing tools and accelerate the adoption of redoxins into synthetic biology applications.

## List of abbreviations

AhpC: alkyl hydroperoxidase subunit C
AhpF: alkyl hydroperoxide reductase subunit F
cpYFP: circularly permuted yellow fluorescent protein.
FAD: flavin adenine dinucleotide
GFP: green fluorescent protein
Gor: glutathione reductase
Gpx3/Orp1: glutathione peroxidase 3
Grx: glutaredoxin
GSH: reduced glutathione
GSSG: oxidized glutathione
IBD: inflammatory bowel disease
iGEM: international genetically engineered machine
KR: KillerRed
Nrd: ribonucleotide reductase
roGFP: redox-sensitive green fluorescent protein
ROS: reactive oxygen species
rxYFP: redox-sensitive yellow fluorescent protein
Tpx: thiol-peroxidase
Trx: thioredoxin
TrxB: thioredoxin reductase
